# Adjunctive Hyperbaric Oxygen Therapy or Alone Antibiotherapy? Methicillin Resistant *Staphylococcus aureus* Mediastinitis in a Rat Model

**DOI:** 10.5935/1678-9741.20150055

**Published:** 2015

**Authors:** Tolga Kurt, Ahmet Vural, Ahmet Temiz, Ersan Ozbudak, Ali Umit Yener, Suzan Sacar, Mustafa Sacar

**Affiliations:** 1 Department of Cardiovascular Surgery, Canakkale Onsekiz Mart University, School of Medicine, Canakkale, Turkey.; 2 Department of Microbiology, Canakkale Onsekiz Mart University, School of Medicine, Canakkale, Turkey.; 3 Department of Cardiology, Canakkale Onsekiz Mart University, School of Medicine, Canakkale, Turkey.; 4 Department of Cardiovascular Surgery, Kocaeli University, School of Medicine, Kocaeli, Turkey; 5 Department of Infectious Disease and Clinical Microbiology, Canakkale Onsekiz Mart University, Canakkale, Turkey.

**Keywords:** Mediastinitis, Hyperbaric Oxygenation, Vancomycin

## Abstract

**OBJECTIVE:**

In the post-sternotomy mediastinitis patients, *Staphylococcus aureus* is the pathogenic microorganism encountered most often. In our study, we aimed to determine the efficacy of antibiotic treatment with vancomycin and tigecycline, alone or in combination with hyperbaric oxygen treatment, on bacterial elimination in experimental S. aureus mediastinitis.

**METHODS:**

Forty-nine adult female Wistar rats were used. They were randomly divided into seven groups, as follows: non-contaminated, contaminated control, vancomycin, tigecycline, hyperbaric oxygen, hyperbaric oxygen + vancomycin and hyperbaric oxygen + tigecycline. The vancomycin rat group received 10 mg/kg/day of vancomycin twice a day through intramuscular injection. The tigecycline group rats received 7 mg/kg/day of tigecycline twice a day through intraperitoneal injection. The hyperbaric oxygen group underwent 90 min sessions of 100% oxygen at 2.5 atm pressure. Treatment continued for 7 days. Twelve hours after the end of treatment, tissue samples were obtained from the upper part of the sternum for bacterial count assessment.

**RESULTS:**

When the quantitative bacterial counts of the untreated contaminated group were compared with those of the treated groups, a significant decrease was observed. However, comparing the antibiotic groups with the same antibiotic combined with hyperbaric oxygen, there was a significant reduction in microorganisms identified (*P*<0.05). Comparing hyperbaric oxygen used alone with the vancomycin and tigecycline groups, it was seen that the effect was not significant (*P*<0.05).

**CONCLUSION:**

We believe that the combination of hyperbaric oxygen with antibiotics had a significant effect on mediastinitis resulting from methicillin-resistant *Staphylococcus aureus*. Methicillin-resistant *Staphylococcus aureus* mediastinitis can be treated without requiring a multidrug combination, thereby reducing the medication dose and concomitantly decreasing the side effects.

**Table:** 

**Abbreviations, acronyms & symbols**	
HBO	= Hyperbaric oxygen
MIC	= Minimum inhibitory concentrations
MRSA	= Methicillin-resistant *Staphylococcus aureus*
PBS	= Phosphate-buffered saline
SD	= Standard deviation

## INTRODUCTION

While mediastinitis developing on the median sternotomy incision after heart surgery is rarely seen (0.5-8%), it is a complication with serious results the majority of the time^[1]^. Hospital stays related to mediastinitis increase hospital costs^[2,3]^. It frequently occurs due to the endogenous flora of the patient inoculating the surgical mediastinal cavity. After cardiac surgery with median sternotomy, the most frequently observed pathogen is methicillin-resistant *Staphylococcus aureus* (MRSA), which is also the bacterium isolated in most blood cultures^[4,5]^.

After diagnosis of mediastinitis, in addition to serious antibiotic treatment, surgical interventions such as primary closure after sternum debridement using vancomycine paste instead of bone vax, closing with a muscle flap after total removal of the sternum using skeletonised left internal thoracic artery and closing the skin completely for closed drainage may be used^[6-8]^. Due to the bactericidal properties of vancomycin on methicillin-resistant staphylococcal infections, systemic vancomycin is the most frequently chosen antibiotic for severe infections like mediastinitis after surgery linked to MRSA^[9]^. However, side effects such as renal failure and hearing loss may be observed to be linked to vancomycin use^[10]^. To protect against these unwanted side effects there is a need to use additional treatments. Adjuvant treatments like hyperbaric oxygen (HBO) treatment are required, but there are insufficient data n this topic in the current literature^[11]^.

Tigecycline is a broad-spectrum glycylcycline effective against Gram-positive and Gram-negative pathogens in soft tissue infections. Between 2004 and 2009, no resistance to tigecycline was observed in soft tissue infections with MRSA, and all the minimum inhibitory concentrations (MIC) were at or below susceptibility break points^[12,13]^.

Studies comparing new antibiotics like tigecycline with vancomycin have shown that tigecycline is more effective against MRSA^[14]^. An experimental rat model found that tigecycline was significantly superior to vancomycin in this application^[15]^. HBO has become a focus of interest for mediastinitis treatment in recent years; however, the patient numbers in both experimental and prospective randomised studies are insufficient^[16]^.

The main aim of our study was to compare the efficacy of HBO alone and combined with tigecycline and vancomycin in an experimental model of mediastinitis. In addition, we aimed to compare the proven therapeutic effectiveness of vancomycin against MRSA with the newer antibiotic tigecycline in an experimental mediastinitis model.

## METHODS

The study was performed at the Canakkale Onsekiz Mart University Experimental Research Application and Research Centre. It was approved by the Canakkale Onsekiz Mart University Animal Research Ethics Committee, Canakkale, Turkey. All animals received human care in compliance with the principles of laboratory animal care developed by the National Academy of Sciences^[17]^. During the study, the animals were housed in Canakkale Onsekiz Mart University Experimental Research Application and Research Centre under veterinary control in rooms with a temperature of 25ºC and humidity of 52%, and given free access to standard feed and water.

### Organisms and susceptibility testing

This study used the ATCC 43300 strain of MRSA. The antibiotic susceptibility was assessed with a Vitek 2 (BioMérieux, Marcy l'Etoile, France) in the Microbiology Laboratory of Canakkale Onsekiz Mart University.

### In vivo rat model and drugs

**Fig. 1:**
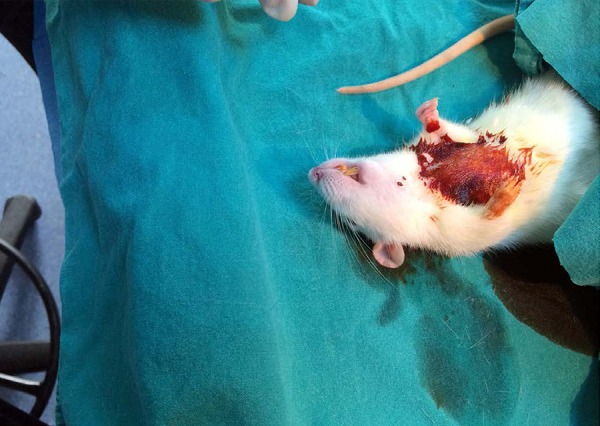
Mediastinal appearance before sternotomy.

This study used 49 female adult Wistar rats weighting 250-300 g, with each group comprising 7 rats. The rats were randomly divided into seven groups, as follows: uncontaminated control (group 1), contaminated control (group 2), vancomycin (group 3), tigecycline (group 4), HBO (group 5), vancomycin+HBO (group 6) and tigecycline+HBO (group 7). To anaesthetise the rats, ketamine hydrochloride (90 mg/kg of body weight; Pfizer, Luleburgaz, Turkey) and xylazine hydrochloride (3 mg/kg of body weight; Bayer AG, Leverkusen, Germany) were used. Each animal was laid securely in supine position for the operation. After, the chest wall was cleaned with 10% povidone-iodine, a sterile towel was used to cover the sternum (Figure 1), and then a central line skin incision was completed. Both major pectoral muscles were divided along the central line and the sternal bone was exposed well. The mid-sternal incision was completed using a no. 15 blade; after, haemostasis was provided by sterile sponges, the mediastinum was entered behind the sternum and a pocket was formed. In the sham group rats, non-contaminated sterile physiological serum was injected into the mediasternal pocket. In all other groups a concentration of 2x10^7^ colony-forming units (CFU)/ml of MRSA strain ATCC 43300 in 1 ml of physiological serum solution was injected into the mediastinum with an insulin injector and the mediastinum was filled with fluid. Later, the sternum and subdermis were sutured with 5/0 polypropylene suture material.

**Fig. 2:**
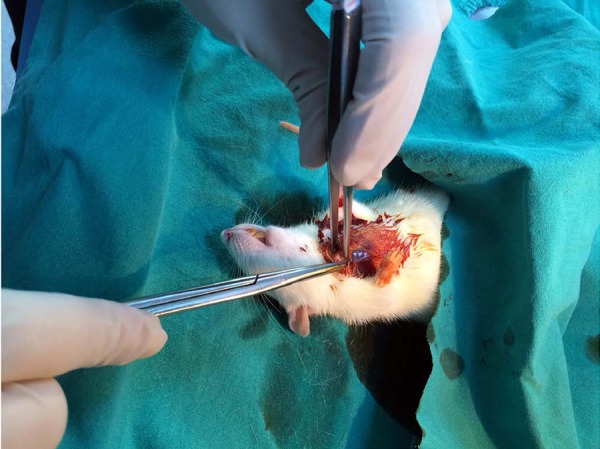
Removing of the upper sternum.

After the surgical procedure, the rats were placed in cages and monitored for hyperaemia, oedema, fever and purulent discharge for seven days. During this time, the rats had free access to standard feed and water. Treatment began 2 days later. In groups 1 and 2, no medication was administered. Postoperatively, in Groups 3, 4, 6 and 7, antibiotic treatment was given twice a day for 7 days. In Groups 5, 6 and 7, HBO with 100% oxygen at a pressure of 2.5 atm was administered once a day for 7 days (Table 1). To identify the microbial load in rats in the non-contaminated and contaminated control groups, at the start of treatment, the non-contaminated animals were sacrificed and equal size thin sections of the upper sternum were removed (Figure 2). The rats in all of the other groups had sternotomy applied 12 h after the end of the 7 day treatment. Equal-sized tissue pieces and the upper section of the contaminated part of the sternum were removed by sternotomy under aseptic conditions. Later, the animals were given a high dose of anaesthetic and sacrificed.

**Table 1:** The results of experimental rat mediastinitis after treatment.

Groups	Therapy	Number of culture negative/ total	Bacterial count: Mean±S.D. log_10_CFU/g
Group 1 n=7	No therapy (Uncontaminated)	10/10	0.0
Group 2 n=7	No therapy (Contaminated)	0/10	3.96±0.56
Group 3 n=7	Vancomycin	0/10	1.90±0.27
Group 4 n=7	Tigesiklin	0/10	2.05±0.32
Group 5 n=7	HBO	0/10	3.11±0.54
Group 6 n=7	Vancomycin+HBO	0/10	1.52±0.95
Group 7 n=7	Tigesiklin+HBO	0/10	1.79±0.98

Mean±S.D. log_10_ CFU/g

### Treatment protocols

Group I (sham group; n=7): Uncontaminated control group, no contamination or antibiotic therapy.

Group II (control group; n=7): Untreated contaminated control group, local contamination with MRSA, no antibiotic therapy.

Group III (vancomycin group; n=7): Vancomycin (Edicine, Sandoz, Istanbul, Turkey) intramuscular injections at 10 mg/kg/ day, twice a day for 7 days.

Group IV (tigecycline group; n=7): Tigecycline (Tygacil, Wyeth Pharmaceuticals, Havant, UK) intraperitoneal injections at 7 mg/kg/day, twice a day for 7 days.

Group V (HBO group; n=7): HBO with 100% oxygen at a pressure of 2.5 atm for 90 min, once a day for 7 days.

Group VI (vancomycin + HBO group; n=7): Vancomycin intramuscular injections at 10 mg/kg/day, twice a day for 7 days, and HBO with 100% oxygen at a pressure of 2.5 atm once a day.

Group VII (tigecycline + HBO group; n=7): Tigecycline intraperitoneal injections at 7 mg/kg/day, twice a day for 7 days, and HBO with 100% oxygen at a pressure of 2.5 atm once a day.

### Assessment of the infection

The explanted sternal upper portion and surrounding tissue samples were placed in sterile tubes and washed in sterile saline solution. Following this, they were placed in tubes containing 10 ml of phosphate-buffered saline (PBS) solution, and vortexed for 1 min to remove the adhering bacteria from the tissue and sternum. Quantification of viable bacteria was performed by culturing serial 10-fold dilutions (0.1 ml) of the bacterial suspension on blood agar plates. All plates were incubated at 37ºC for 48 h and evaluated for the presence of MRSA. The organisms were quantitated by counting the number of CFU/g per tissue. The results were converted to log form.

### Statistical analysis

Quantitative culture results are presented as arithmetic mean±standard deviation (SD) CFU/ml. Differences among the groups were evaluated using Kruskal-Wallis analysis and multiple comparisons between the groups were performed using the Mann-Whitney test. Differences were considered statistically significant when *P*<0.05. Data were analysed by statistical software (SPSS for Windows 17.0; SPSS, Chicago, IL).

## RESULTS

In this study, we aimed to assess the efficacy of vancomycin, which has been proven for the treatment of mediastinitis, in comparison with the relatively newly used tigecycline, HBO and combinations of these. In all contaminated rats, macroscopic mediastinitis findings were observed (tissue oedema). Especially in the untreated contaminated group, advanced-degree infected tissues with purulent fibrin adhesions were present. With the exception of group 1, comprising non-contaminated rats which were not given antibiotherapy, macroscopic mediastinitis findings in the groups were compared. The culture-negative rates and bacterial counts linked to mediastinitis in each group are shown in Table 1. The mean growth sizes of microorganisms in the mediastinal areas within the sternum were compared. When the quantitative bacterial counts in the mediastinal region within the sternum in the contaminated non-treated group (group 2) were compared with the treated groups (group 3-7), a significant reduction was observed (*P*<0.05). Comparing groups with vancomycin and tigecycline administered as prophylactics, there was no statistically significant difference observed (*P*>0.05). However, comparing the antibiotic groups with the same antibiotic combined with HBO, there was a significant reduction in the number of microorganisms (*P*<0.05). In addition, when the vancomycin+HBO (group 6) and tigecycline+HBO (group 7) were compared, there was a significant reduction in the amount of microorganisms in the group treated with combined vancomycin (*P*<0.05).

Comparing the use of HBO alone with the untreated contaminated group, a significant reduction in bacteria counts was observed (*P*<0.05); however, comparing HBO alone with the vancomycin and tigecycline groups, the effect was observed not to be significant (*P*>0.05).

## DISCUSSION

In this experimental mediastinitis model, we aimed to compare tigecycline with vancomycin, which is classically used to treat mediastinitis linked to MRSA. In addition, we aimed to compare the treatment efficacy of both antibiotics combined with HBO. We proved that there was no statistical superiority of either antibiotic when used alone.

In this study, we used previously applied experimental rat mediastinitis models^[18,19]^. When the literature was examined, we found several animal models using tigecycline, vancomycin and HBO against MRSA, so we aimed to use these antibiotics and HBO. However, we did not discover any rat model involving the combined use of tigecycline and HBO^[15,20-22]^. Tigecycline was first derived from minocycline, and is a broad spectrum antibiotic of the glycylcycline class with broad in vitro activity^[23,24]^.

There is no requirement to regulate the dose of tigecycline for patients with renal failure or even those with mild to moderate hepatic dysfunction; moreover, it is not induced or inhibited by cytochrome p450^[23,25]^. In addition, studies of medication interactions have shown that when tigecycline is used with medications like warfarin and digoxin, no side effects were observed in healthy individuals^[26,27]^. The most frequently observed side effects are mild to moderate nausea and vomiting in the early stages, mainly the first 1-2 days^[28]^.

Tigecycline has been used successfully in the treatment of many severe and mortally progressing types of mediastinitis, such as *Mycobacterium chelonae*, pan-resistant *Acinetobacter baumannii* mediastinitis and multidrug-resistant *Klebsiella pneumoniae* mediastinitis^[29-31]^.

Vancomycin is an antibiotic frequently chosen for the treatment of severe MRSA infections like mediastinitis. As this has a slow killing effect on glycopeptide bacteria, it should be used for treatment of long-term diseases and this may cause a risk of severe kidney failure for patients^[32-34]^. Although vancomycin is not routinely recommended for use in cardiac surgery, it is important to use it in patients with an increased risk or cases with a high incidence of MRSA^[35]^. However, there has been no study on the role of tigecycline in a rat model or in combination with HBO.

Another aim of our study was to compare the efficacy of HBO combined with antibiotic treatment or used alone for MRSA mediastinitis. HBO therapy involves the treatment of patients with 100% oxygen at higher than atmospheric pressure within a hyperbaric chamber^[36]^. HBO causes increased dissolved oxygen levels in blood; this in turn causes hyperoxia in tissues. It restores the bactericidal capacity of leukocytes in hypoxic wounds by increasing tissue oxygen tensions^[37]^. Moreover, it has been used to treat difficult cases for many years. Although the effective mechanism of HBO is not fully understood, it has been observed to activate many mechanisms, including collagen synthesis^[38]^, accelerated macrophage infiltration^[39]^, fibroblast proliferation^[40,41]^, growth factor proliferation^[42,43]^, modulation of hypoxia-inducible factor 1 alpha^[44]^, improved antibactericidal capacity^[45]^, bacteriostatic effects^[46,47]^ and stimulation of angiogenesis^[48]^. In this study, when the use of HBO alone was compared with the untreated group with MRSA mediastinitis, a reduction in the number of bacteria was found; however, when HBO alone was compared with vancomycin and tigecycline, the effect was not observed to be significant.

HBO (100% oxygen once per day for 90-120 min at 2-3 atm) can generally be combined with antibiotics like penicillins, beta-lactamase inhibitors, cephalosporins, aztreonam, imipenem, vancomycin, clindamycin, rifampin, aminoglycosides, fluoroquinolones, trimethoprim/sulphamethoxazole, metronidazole, teicoplanin, quinupristin and dalfopristin, and is used to treat osteomyelitis. Thus, the efficacy of an antibiotic is increased in hypoxic tissues^[49]^. It has been observed that HBO increases the effectiveness of debridement and antibiotic treatment. Topuz et al.^[50]^ observed heightened efficacy when HBO was combined with antibiotics and surgery for spinal tuberculosis patients.

In our study, similar to other research using HBO and combinations of a classic and new antibiotic like vancomycin and teicoplanin, we researched the efficacy of these treatments in rats with MRSA mediastinitis. While HBO alone resulted in a significant drop in the number of bacteria, its combination with either antibiotic significantly increased the efficacy of the medication.

When this study is evaluated, there are some limitations to consider. The pathogenic bacteria were directly inoculated to the sternal and mediasternal layers, and this situation is not appropriate for clinical comparison. For ethical reasons, the treatment continued for 7 days, but longer duration of treatment may produce better results. For technical reasons, we did not obtain sufficient data on systemic infection (direct histological examination, white blood cells). Moreover, we did not obtain sufficient data on the serum antibiotic concentrations. In spite of these limitations, this is the only study to compare the effects of HBO with new generation antibiotics like tigecycline in the in vivo environment.

## CONCLUSION

We conclude that the combination of HBO with antibiotics shows a significant effect on MRSA mediastinitis. HBO can also reinforce antibacterial therapy and speed up healing. MRSA mediastinitis can be treated without requiring multidrug combinations, thereby decreasing the required dose of medication, and as a result, reducing its side effects. In the future, ethical permission for human studies will determine the efficacy of HBO in the treatment of MRSA mediastinitis.

**Table:** 

**Authors' roles & responsibilities**	
TK	Final manuscript approval
AV	Study conception and design; final manuscript approval
AT	Manuscript writing and critical review of its content; final manuscript approval
EO	Analysis/interpretation of data; statistical analysis; final manuscript approval; manuscript writing or critical review of its content
AUY	Conduct of operations/experiments; final manuscript approval
SS	Manuscript writing and critical review of its contents; final manuscript approval
MS	Study conception and design; final manuscript approval
